# Influence of orthodontic appliance-derived artifacts on 3-T MRI movies

**DOI:** 10.1186/s40510-018-0204-6

**Published:** 2018-02-19

**Authors:** Erika Ozawa, Ei-ichi Honda, Kulthida Nunthayanon Parakonthun, Hiroko Ohmori, Kazuo Shimazaki, Tohru Kurabayashi, Takashi Ono

**Affiliations:** 1 Department of Orthodontic Science, Tokyo Medical and Dental University (TMDU) Graduate School, 1-5-45 Yushima, Bunkyo-ku, Tokyo, 113-8549 Japan; 2 Department of Oral and Maxillofacial Radiology, The University of Tokushima, Tokushima, Japan; 3 Department of Pedodontics & Preventive Dentistry, Srinakharinwirot University, Bangkok, Thailand; 4 Department of Oral and Maxillofacial Radiology, Tokyo Medical and Dental University (TMDU) Graduate School, Tokyo, Japan

## Abstract

**Background:**

Magnetic resonance imaging (MRI) has been used to study configurations of speech organs in the resting state. However, MRI is sensitive to metals, and numerous types of metallic appliances, most of which have a large magnetic susceptibility, are used in orthodontic treatment and may cause severe artifacts on MRI. We have developed techniques for obtaining MRI movies of the oral region, to evaluate articulatory changes, especially movement of the tongue, palate, and teeth, pre- and post-orthodontic/orthognathic treatment. We evaluated the influence of artifacts caused by orthodontic appliances, including fixed retainers, metal brackets, and wires, on measurements in 3-T MRI movies.

**Methods:**

Sixteen healthy young adults (nine males, seven females; average age, 27 years) with normal occlusion were recruited. Four types of customized maxillary and mandibular plates were prepared by incorporating one of the following into the plate: (a) nothing, (b) a fixed canine-to-canine retainer, (c) metal brackets for the anterior and molar teeth, or (d) clear brackets for the anterior teeth and metal brackets for molars. A 3-T MRI movie, in segmented cine mode, was generated for each plate condition while participants pronounced a vowel-consonant-vowel syllable (/asa/). The size of the artifact due to the metallic brackets was measured. The face size and orthodontically important anatomical structures, such as the velum, the hard palate, and the laryngeal ventricle, were also measured.

**Results:**

A large artifact was observed over the entire oral region around orthodontic appliances, altering regional visibility. The velopharyngeal height was measured as significantly longer in the presence of metal brackets. The maximum artifact size due to a metallic bracket was > 8 cm. Our results show that even if it is possible to obtain the measurements of palate length, nasion to sella, and nasion to basion in individuals wearing metal brackets for molars, the measurements might be affected due to the presence of artifacts.

**Conclusions:**

Orthodontic appliances, including metallic materials, sometimes produce significant measurement error in speech evaluation using MRI movies, which often become invisible or distorted by metallic orthodontic appliances. When the distorted image is measured, caution should be exercised, as the measurement may be affected. Based on the study, it is concluded that orthodontists should not necessarily remove all metallic appliances before MRI examination because the influence varies among the appliances and should also know that a significant measurement error in speech evaluation using MRI movie may occur by image distortion caused by metallic artifacts.

## Background

Magnetic resonance imaging (MRI) produces images of soft tissues with higher contrast resolution than computed tomography (CT), without radiation exposure, and various MRI software techniques and hardware have been developed. This technique has been applied to assess the dynamic movement of the heart. Moreover, by combining several technologies, observation of the movement of the speech organ also became possible [1–4].

At present, many heart studies [5] and some speech organ studies [6] have been performed using MRI movies, but delineation of hard tissues, including the bone and teeth, remains difficult, although conventional methods for teeth delineation in MRI have been reported [7–9]. Our group researches MRI movie techniques for oral lesions, to evaluate articulation changes, especially movement of the tongue, palate, and teeth, before and after orthodontic and oral surgical treatment [10] and have reported many relevant findings [11–13].

Articulation disorders are caused by various factors, including congenital deformation, malocclusion, developmental maxillofacial disturbances, and tongue habits. Orthodontics is used to treat these conditions. Treatment starts in childhood, in many cases, and radiographic imaging is frequently used. For protection from radiation, patient exposure should be reduced as much as possible, especially in children. MRI is therefore suitable because it does not require the use of radiation, and MRI movies are the best approach to observe articulation over an extended period. Nevertheless, MRI is more sensitive to metals than radiography, and metallic artifacts caused by metallic fillings, such as crowns or inlays often appear in the oral maxillofacial region, complicating diagnosis. Moreover, numerous types of metallic appliances are used in orthodontic treatment and most have a large magnetic susceptibility, leading to severe artifacts on MRI. Although it is ideal to remove all metallic appliances before MRI examination, debonding and rebonding may harm the tooth enamel and extend the treatment period [14]. Knowledge of the effect of various orthodontic appliances on MRI can circumvent unnecessary removal of orthodontic appliances and is important for orthodontists and radiologists.

The aim of this study was to examine the influence of orthodontic appliance-derived artifacts on MRI movie images and to evaluate their influence on diagnosis. The null hypothesis was that the orthodontic metallic appliances do not lead to artifacts in MRI examinations.

## Methods

### Subjects

Nine healthy males and seven healthy females with normal occlusion were recruited. The mean age of the male group was 26.8 ± 0.6 (mean ± standard deviation [SD]) years, and that of the female group was 26.3 ± 0.5 years. Males and females did not significantly differ with respect to overjet, overbite, upper arch length, or lower arch length (Table 1). Written informed consent was obtained from all 16 subjects prior to their participation in the study. All procedures in this study were approved by the Ethics Committee (No. 1282) of Tokyo Medical and Dental University and complied with the Code of Ethics of the World Medical Association (Declaration of Helsinki).

**Table 1:** Sex differences in occlusal condition

	Male (*N* = 9)	Female (*N* = 7)	Significance
Overjet (mm)	1.9 ± 1.5	2.6 ± 0.8	NS
Overbite (mm)	2.2 ± 1.1	2.9 ± 0.9	NS
Upper arch length (mm)	48.3 ± 3.3	45.4 ± 2.6	NS
Lower arch length (mm)	44.4 ± 2.8	43.3 ± 2.5	NS

Mean ± standard deviation

*NS* not significant

### Description of procedures

**Fig. 1:**
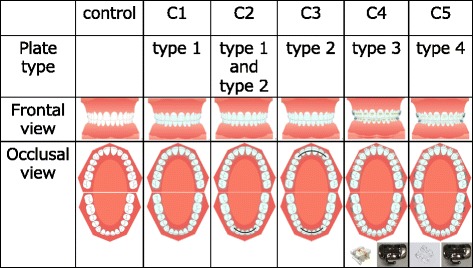
Schematic diagrams of six conditions with four types of customized plates. Insets depict the brackets for anterior and molar teeth that are incorporated in each type of plate. In C2, the maxillary plate is type 1, and the mandibular plate is type 2

Four types of customized maxillary (Mx) and mandibular (Md) plates made of Biostar® (DURASOFT PD, Rocky Mountain Morita, Tokyo, Japan) were prepared (Fig. 1). The plate types were divided as follows: nothing incorporated in the plate (type 1), a fixed canine-to-canine retainer (HI-T™ II TWIST-WIRE, 0.015" (0.381-mm) in diameter, 3M, Tokyo) incorporated in the plate (type 2), 0.018" (0.4572-mm)-slot metal brackets (CLEAR BRACKET SL+, Dentsply, Tokyo) for anterior teeth and metal brackets for molars (OPA-K, TOMY INTERNATIONAL INC., Tokyo) incorporated (type 3), and 0.018”-slot clear brackets (CrystaBrace3, Dentsply, Tokyo) for anterior teeth and metal brackets for molars (OPA-K, TOMY INTERNATIONAL INC.) incorporated (type 4; Fig. 1). No archwire was applied.

### Magnetic resonance imaging of subjects

A 3-Tesla (3-T) MRI scanner (Magnetom Spectra, Siemens, Munich, Germany) was used, and movie images were taken following the method reported by Nunthayanon and colleagues [15]. The mid-sagittal plane was imaged using the following parameters: TR = 22.5 ms, echo time (TE) = 2.07 ms, field of view = 256 × 256 mm, pixel size = 1 × 2 mm, slice thickness = 4 mm, and the total acquisition time was 24 s.

### Speech task and speech condition

**Fig. 2:**
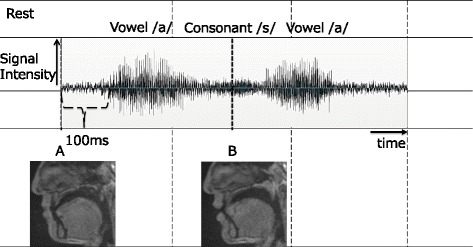
A representative sound wave and timing. Signals obtained at the rest stage of 100 ms before production of the first vowel (stage A) and at the middle of production of the consonant (stage B) were analyzed

The subjects were required to repeat the vowel-consonant-vowel (VCV) syllable (/asa/) 16 times at 1500-ms intervals. The consonant was chosen because a previous study showed that during production of /s/, the velopharyngeal structures are not affected by upright or supine body position [12]. Subjects were scanned under six conditions with the plate: (1) neither Mx nor Md (control), (2) type 1 for both Mx and Md (C1), (3) type 1 for Mx and type 2 for Md (C2), (4) type 2 for both Mx and Md (C3), (5) type 3 for both Mx and Md (C4), and (6) type 4 for both Mx and Md (C5; Fig. 1). The subjects were not aware of the plate type used in the experiment. Recorded sound data were manipulated by Kulthida’s method [15]. Sound stage was determined by the manipulated sound data. Images obtained at the rest phase (100 ms before pronunciation; stage A) and the consonant phase (the middle of consonant /s/; stage B) were chosen for analysis (Fig. 2).

### Definition of measurements

**Fig. 3:**
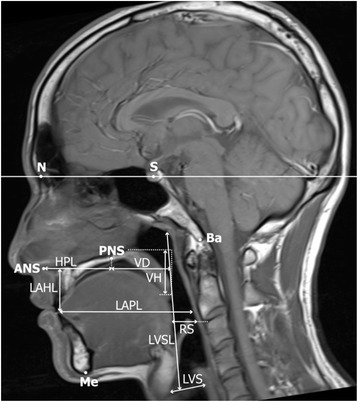
Reference lines. Eight white lines were determined based on the study by Perry [15]. Sella-Nasion (SN) was used as the standard, and eight linear measurements are shown. Velar depth = VD, velar height = VH, retroglossal space = RS, laryngeal ventricle space = LVS, laryngeal ventricle-sphenoid sinus length = LVSL, hard-palate length = HPL, lingual apex-pharyngeal length = LAPL, lingual apex-hard palate length = LAHL. And six measurements for face size. N = Nasion, Ba = Basion, S = Sella, ANS = anterior nasal spine, PNS = posterior nasal spine, Me = Menton

Twelve images of stages A and B were obtained in the six conditions. Eight linear parameters were determined based on the study by Perry [12] (Table 2, Fig. 3). The standard line (Sella-Nasion [SN] reference) was based on the T1-weighted image by turbo spin echo (TSE) sequence. Five facial measurements were also performed on the TSE image by Perry (Table 3 and Fig. 3). There were significant sex differences in two parameters (Nasion to Sella and face height; Table 5; *p* < 0.01). The measurements were performed using Osirix MD (Pixmeo Sarl, Geneva, Switzerland).

**Table 2:** Definition of linear measurements

Linear measurement	Definition
Velar depth (VD)	Length of velar on the plane parallel to Sella-Nasion (SN) reference
Velar height (VH)	Length of velar on the plane parallel to vertical reference to SN reference
Retroglossal space (RS)	Distance between the top and bottom of the pharyngeal wall on the plane parallel to vertical reference to SN reference
Laryngeal ventricle space (LVS)	Distance between the point of the most swelling to the point of the most remaining in the background
Laryngeal ventricle-sphenoid sinus length (LVSL)	Distance between the posterior point of the larynx ventricle and the bottom of the sphenoid bone
Hard-palate length (HPL)	Distance between the anterior nasal spine (ANS) and the posterior nasal spine (PNS)
Lingual apex-pharyngeal length (LAPL)	Distance between the lingual apex and the posterior pharyngeal wall on the plane parallel to SN reference
Lingual apex-hard palate length (LAHL)	Distance between the lingual apex and the hard palate wall on the plane parallel to vertical reference to SN reference

**Table 3:** Sex differences in face size

	Male (*N* = 9)	Female (*N* = 7)	Significance
Nasion to Sella (mm)	67.6 ± 5.6	63.0 ± 2.9	*p* < 0.01
Nasion to Basion (mm)	102.9 ± 4.4	89.1 ± 10.9	NS
Face height^a^ (mm)	126.3 ± 10.3	114.9 ± 3.0	*p* < 0.001
Palate length^b^ (mm)	38.9 ± 6.3	34.7 ± 5.2	NS
PNS to Basion (mm)	55.4 ± 5.7	53.9 ± 5.6	NS

Mean ± standard deviation

*NS* not significant

^a^Nasion to Menton

^b^ANS to PNS

### Magnetic resonance imaging with orthodontic appliances

Each bracket was placed on a pole in an acrylic cylinder container (diameter: 4.5 mm) filled with water. The sagittal, coronal, and transverse direction images were obtained by TSE and gradient-echo (FLASH). The range of artifacts surrounding each bracket was evaluated based on the method by Imai et al. [16].

### Subtraction imaging

Four kinds of subtracted images were generated. First, the control image was subtracted from the C1 image to confirm similarity. Because there was no clear difference, the C1 image was defined as a standard, and three kinds of subtraction imaging (C1–C3, C1–C4, and C1–C5) were performed. Paired images were visually aligned with the reference to an outline of the skull and cropped to the same size by customized software (Image Rugle 2009 Medic Engineering Inc., Kyoto, Japan). After processing, the images were subtracted by Adobe Photoshop CS6 software (Adobe Systems Inc., San Jose, CA).

### Statistical analysis

Each organ on MRI movie images was measured three times over 5 days. A single examiner (EO) performed measurements to avoid interobserver error. Intraobserver reliability was assessed by intraclass correlation coefficients (ICC). The measurement errors determined by ICC were very small (range: *p* < 0.05), indicating the measurements were reproducible. Analysis of variance (ANOVA) was used to evaluate the difference of the length between stages A and B and among each condition. Dunnett’s test, for multiple comparisons between the control and each condition, was used when a significant difference was observed. Significance level was defined as *p* < 0.05. With regard to velar size, the logistic analysis of the relation between the number of measurable cases and the face size in C4 and C5 was used. The analysis was performed in two dimensions of the velar depth (VD) and velar height (VH), given that the velum plays an important role in pronunciation.

## Results

**Fig. 4:**
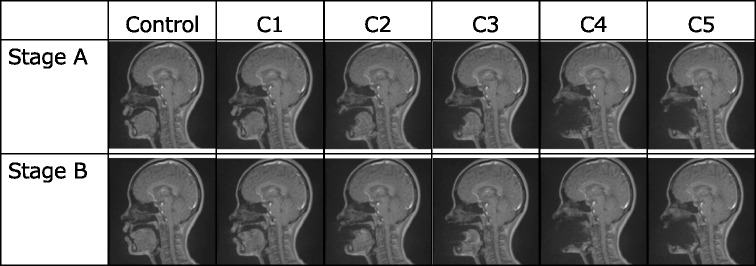
Magnetic resonance movie images in six conditions at stages A and B

Typical MRI movie images taken in the six conditions are shown in Fig. 4, and representative sound data combined with the image of subject are shown in Fig. 2. At stage A, the articulators were in the rest position, in which the velum was placed on the posterior aspect of the tongue. At stage B, the tongue actively moved anteriorly toward the premaxillary area. No artifact was observed in C1 at either stage. In C2 and C3, clear artifacts were observed around the lips and incisors, and the surrounding soft tissues disappeared. In C4 and C5, more severe artifacts were observed all over the oral cavity, and the tongue and palate disappeared completely.

The number of measurable cases is shown in Tables 4. The number in both stages was similar. All distances could be measured in C1 at both stages. A significant sex difference was observed in RS in C4 and C5 at stage A and in RS in C4 at stage B (*p* < 0.05).

**Table 4:** Number of cases with measurable parameters at stages A and B in five conditions (*N* = 16)

		C1	C2	C3	C4	C5
VD	Stage A Stage B	16 (9) 16 (9)	16 (9) 16 (9)	16 (9) 16 (9)	4 (4) 4 (4)	6 (5) 7 (5)
VH	Stage A Stage B	16 (9) 16 (9)	16 (9) 16 (9)	16 (9) 16 (9)	4 (4) 5 (5)	6 (5) 7 (6)
RS	Stage A Stage B	16 (9) 16 (9)	16 (9) 16 (9)	16 (9) 16 (9)	6 (6) 5 (5)	6 (6) 7 (6)
LVS	Stage A Stage B	16 (9) 16 (9)	16 (9) 16 (9)	16 (9) 16 (9)	16 (9) 16 (9)	16 (9) 16 (9)
LVSL	Stage A Stage B	16 (9) 16 (9)	16 (9) 16 (9)	16 (9) 16 (9)	16 (9) 16 (9)	16 (9) 16 (9)
HPL	Stage A Stage B	16 (9) 16 (9)	12 (7) 11 (6)	5 (3) 5 (3)	0 (0) 0 (0)	0 (0) 0 (0)
LAPL	Stage A Stage B	16 (9) 16 (9)	0 (0) 0 (0)	0 (0) 0 (0)	0 (0) 0 (0)	0 (0) 0 (0)
LAHL	Stage A Stage B	16 (9) 16 (9)	0 (0) 0 (0)	0 (0) 0 (0)	0 (0) 0 (0)	0 (0) 0 (0)

Number of females depicted in parentheses

Abbreviations: see Table 2

Regional visibility was different between the fixed retainer and the orthodontic brackets (Table 5). In the fixed retainer condition, only the anterior part of the tongue was invisible in all cases. Although the velopharynx, the posterior part of the tongue, and the vocal fold were clearly visible, the hard palate (HPL) was occasionally visible. In contrast, with the orthodontic brackets, most organs were invisible; only the vocal fold was always clearly visible.

**Table 5:** Visibility of each organ in subjects wearing orthodontic appliances

	Fixed retainer	Brackets
Velopharynx	○	△
Hard palate	△	×
Anterior part of tongue	×	×
Posterior part of tongue	○	×
Vocal fold	○	○

Symbols: ○, clearly visible; △, occasionally visible; ×, completely invisible

**Fig. 5:**
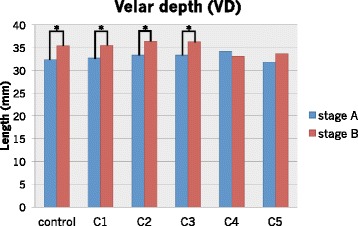
The length of VD in six conditions at stages A and B

**Fig. 6:**
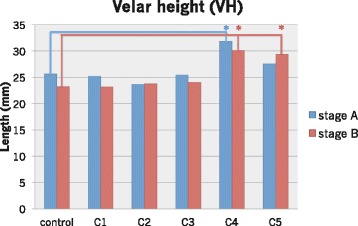
The length of VH in six conditions at stages A and B

The eight length measurements where significant differences were observed at stages A and B in the six conditions are shown in Figs. 5 and 6. Only the VH in C4 at stage A and in C4 and C5 at stage B was significantly longer than that of the control, because the upper margin was located superiorly (*p* < 0.05). It was impossible to measure the HPL in C4 and C5 and the lingual apex−pharyngeal (LAPL) and lingual apex−hard palate (LAHL) in C2–C5 because anatomical structures disappeared due to the severe artifacts.

**Fig. 7:**
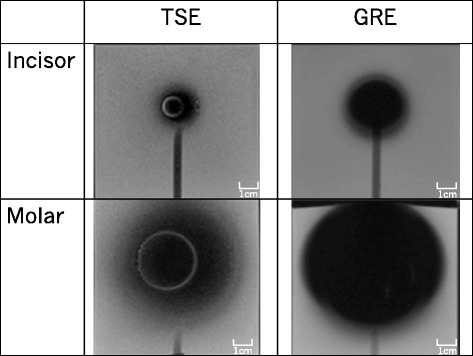
Magnetic resonance images of incisor and molar brackets taken with turbo spin echo (TSE) and gradient echo (GRE) sequences

**Fig. 8:**
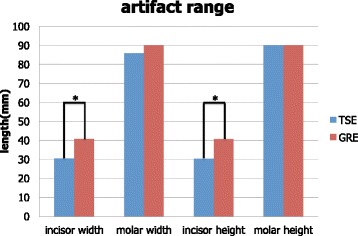
Comparison of the range of incisor and molar bracket-derived artifact between two magnetic resonance imaging sequences

Typical MRI images of the brackets for the anterior and molar teeth are shown in Fig. 7, and the measurements are shown in Fig. 8. The most severe artifact was observed in a gradient echo image involving a molar bracket. The artifact size was greater than 8 cm in diameter. Parameters of the brackets for the incisor could be measured, but most parameters of the molar bracket could not be measured. There was a significant difference between turbo echo and gradient echo images in the incisor bracket.

There was no significant difference in any of the items (Tables 6). However, there was a tendency for an increase in the length of the Nasion (N) to Basion (Ba) and the palate length (ANS to PNS), which corresponded with the increase in the number of measurable cases.

**Table 6:** The relationship between number of measurable cases and individual linear measurements in C4 and C5 at rest and the consonant

	VD	VH		
	C4	C5	C4	C5
Nasion to Sella rest the consonant	NS NS	NS *p* < 0.1	NS NS	NS *p* < 0.1
Nasion to Basion rest the consonant	NS NS	*p* < 0.1 NS	NS NS	*p* < 0.1 NS
Face height rest the consonant	NS NS	NS NS	NS NS	NS NS
Palate length rest the consonant	*p* < 0.1 *p* < 0.1	NS *p* < 0.1	*p* < 0.1 *p* < 0.1	NS *p* < 0.1
PNS to Basion rest the consonant	NS NS	NS NS	NS NS	NS NS

*p* > 0.1

*NS* not significant

**Fig. 9:**
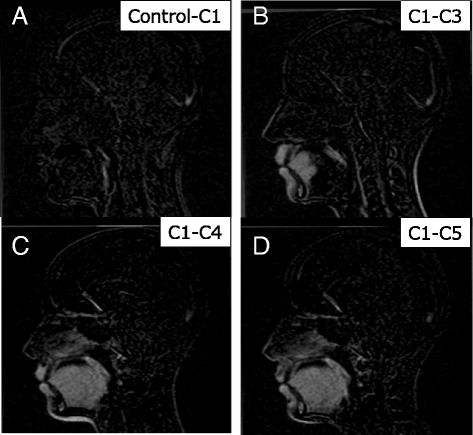
Subtracted images. **a** Subtraction of control from C1; **b** subtraction of C1 from C3; **c** subtraction of C1 from C4; **d** subtraction of C1 from C5

Figure 9 shows the subtracted images (orthodontic appliance vs. no orthodontic appliance). The lips and the anterior part of the tongue were affected by the fixed retainer. Artifacts from the brackets affected the lips, the whole tongue, and the chin, while the larynx was not affected.

## Discussion

### The measurable range in subjects wearing orthodontic metallic appliances

The severity of artifacts mainly depends on the size, shape, and magnetic susceptibility of the metal of the orthodontic appliance [17]. The influence of metallic materials used in orthodontic treatment on MRI has been reported in a number of studies [18–20]. Nonetheless, no studies have reported quantitative data of the artifact range generated by an orthodontic bracket, including the range of organs influenced by the artifact.

In our study, the null hypothesis was rejected: the orthodontic metallic bracket for the molars used in this study caused a very severe artifact. The results indicated that tissues located within a 5-cm radius from a bracket could be influenced by an artifact, and observation and measurement of organs and tissues in this range would be distorted. The tongue, HPL, velopharynx, cervical vertebrae, epiglottis, and hyoid bone are included in the area, and the range could be clearly seen in the subtraction images. Thus, caution should be exercised when measuring the length of organs in patients wearing orthodontic appliances during MRI. In terms of the type of metallic bracket, there was a significant difference in artifact size between brackets used on the anterior and on the molar teeth, probably due to the weight and composition of the brackets. The metallic weights of the anterior and molar brackets are approximately 0.01 and 0.1 g, respectively. While both types of brackets are made of stainless steel, the crystal structure of stainless steel in the anterior bracket is austenitic and that of the molar bracket is martensitic. Many studies have reported that martensitic stainless steel has markedly higher magnetic susceptibility than austenitic stainless steel [21–23]. Thus, the molar brackets caused an immense artifact because of its heavier weight and higher magnetic susceptibility.

### Measurable range due to individual difference

Since metallic artifacts caused image distortion, it was difficult to measure the target region influenced by the artifact accurately. Despite a clear metallic artifact, the length of the velum was measurable in some cases. There was no significant sex difference in the length of the palate (Table 3). However, the upper pharyngeal space in males was wider than that in females. No reports discuss this difference, and future research on this topic is necessary.

During general pronunciation, articulatory organs, such as the tongue and the soft palate, change form. By observing these organs in MRI movie images during pronunciation of /asa/, we found that the velar apex was elevated with mouth opening and contacted the posterior wall of the pharynx. In some subjects, the velar apex did not contact the posterior pharyngeal wall. In contrast, the velar apex in all subjects contacted the posterior pharyngeal wall during production of /s/, and velopharyngeal closure was completed. The tongue apex also approached the lingual surface of the maxillary anterior teeth.

When returned to the position for production of /a/, the tongue blade sagged, and the velar slowly separated from the posterior wall of the pharynx. In phonetics, the velopharyngeal closure is completed by the postero-superior elevation of the velum, the contraction of the pharyngeal constrictor muscles, and the formation of Passavant’s ridge toward the pharyngeal cavity. The velopharyngeal closure is sometimes not completed during production of /a/.

The velar apex is elevated by the construction of the levator veli palatini muscle and changes form. Production of /s/ is carried out by the formation of a narrow space between the tongue apex and the lingual side of the maxillary anterior teeth [24, 25]. Since the movement of the tongue as well as the velum is related to pronunciation, orthodontic concerns, such as overjet and overbite, which determine tongue position, influence pronunciation. The tongue approaches the lingual side of the maxillary anterior teeth on production of /s/. Excessive overjet increased the length of LAPL and RS in this study, whereas the values of LAPL and RS showed no change, because there was no significant difference in overjet.

A tongue-thrusting habit influences pronunciation, and the tongue position and the RS sometimes change [26]. Future studies will be able to diagnose tongue-thrusting habit by using MRI movies.

### Image distortion

The velopharynx was rostrocaudally prolonged by metallic artifacts. The subtraction image showed that the artifact spread in the direction from the head to the mandible. Although there was a significant difference in the VD between the rest phase (i.e., stage A) and the consonant phase (i.e., stage B) in control, C1, C2, and C3, this difference was not observed in C4 and C5. This may be due to image distortion by metallic artifacts from the molar brackets. The VH in C4 and C5 was significantly longer than that in control. This difference may be attributable to the weight of the metal. As a metal was included in the anterior and premolar brackets as well as in the molar brackets in C4, the size of the artifact in C4 would increase. In terms of the VH, during production of /s/, the velum partially contacted the HPL, and the tip was pushed to the posterior wall of the pharynx. The velar shape became vertically longer and was easily influenced by a metallic artifact caused by premolar and molar brackets in C4 and C5. Consequently, the measurement was altered, differing from that in the control. In some cases, the velar length could not be measured because of the metallic artifact. Whether the velar length is measurable depends on face size, because the range of orthodontic appliance-derived artifacts is almost constant. In this study, there was no clear relation between face size and measurable cases, but a trend was noted for the length of N to Ba and ANS to PNS. This indicates that as the antero-posterior velar length increases, the probability of measurable cases also increases. Phonetic analysis of articulatory movements by MRI movie is difficult in children who wear orthodontic appliances because the face is small, and the articulators are easily influenced by metallic artifacts. In contrast, orthodontic appliance is not always a problem in patients with larger faces.

## Conclusions

Orthodontic appliances, including metallic materials, may produce a significant error in measurement of the articulatory organs when using MRI movie images. Metallic orthodontic appliances such as the fixed retainer and metal brackets often make anatomical structures disappear and become distorted by signal void caused by metallic artifact. When the velum is evaluated in a distorted image, caution should be exercised as the measurement may be altered. In conclusion, the influence on MRI examination varies among orthodontic metallic appliances and orthodontists should not necessarily remove all metallic appliances before MRI examination. Meanwhile, orthodontists should know the fact that a significant measurement error in speech evaluation using MRI movie may occur by image distortion caused by metallic artifacts and share the information with radiologists.
